# Nitric oxide synthase, Calcitonin Gene-Related Peptide and inflammatory mechanisms are involved in GTN induced neuronal activation

**DOI:** 10.1186/1129-2377-1-S14-P217

**Published:** 2013-02-21

**Authors:** R Ramachandran, K Bhatt, I Jansen-Olesen, J Olesen

**Affiliations:** 1 Glostrup Hospital, Denmark

## Introduction and objective

Infusion of glyceryl trinitrate (GTN), a nitric oxide (NO) donor in awake freely moving rats closely mimics a universally accepted human model of migraine and responds to sumatriptan treatment [1,2]. Here we analyse the effect of nitric oxide synthase (NOS) and calcitonin gene-related peptide (CGRP) systems on the GTN induced neuronal activation in this model.

## Methods

The femoral vein was catheterized and rat allowed recovering for ten days before infusion of GTN (4 µg/kg/min, for 20 min, i.v.). Immunohistochemistry was used to measure Fos, nNOS and CGRP protein expression. Western blot was done to re-confirm the nNOS expression. Olcegepant (1 mg/kg) for 3 mins was given both as a pre-treatment and post treatment to analyse its effect on Fos activation. The response to pre-treatment with L-NAME (40 mg/kg) and NK-1 antagonist, L-733060 (1mg/kg) was also measured at the activation level.

## Results

GTN treated rats showed a significant increase of nNOS and CGRP in dura and CGRP in trigeminal nucleus caudalis (TNC). Upregulation of the nociceptive marker Fos was observed in TNC at 2 and 4 hrs after the infusion. The activation at 4 hrs was inhibited by pre-treatment with olcegepant. However, post treatment with olcegepant could not inhibit this activation. Pre-treatment with L-NAME and L-733060 also significantly inhibited the GTN induced Fos expression.

## Conclusion

The present study indicates that inhibition of CGRP, NOS and inflammatory systems all block GTN induced neuronal activation. These findings also predict that pre-treatment with olcegepant may be a better option than post-treatment to study inhibitory effect on GTN migraine models.
